# Successful treatment for sorafenib-induced liver dysfunction: a report of case with liver biopsy

**DOI:** 10.1186/s40792-016-0131-z

**Published:** 2016-01-14

**Authors:** Daisuke Kuroda, Hiromitsu Hayashi, Hidetoshi Nitta, Katsunori Imai, Shinya Abe, Daisuke Hashimoto, Akira Chikamoto, Takatoshi Ishiko, Toru Beppu, Hideo Baba

**Affiliations:** Department of Gastroenterological Surgery, Graduate School of Medical Sciences, Kumamoto University, 1-1-1 Honjo, Kumamoto, 860-8556 Japan

**Keywords:** Hepatocellular carcinoma, Sorafenib, Liver dysfunction, Hyperbaric oxygen therapy

## Abstract

Sorafenib is an oral multikinase inhibitor with anti-proliferative and anti-angiogenic effects and is used worldwide for the treatment of advanced or metastatic hepatocellular carcinoma (HCC). While the significant survival benefit of sorafenib in patients with advanced HCC was demonstrated, various treatment-related adverse events might happen. Of them, the incidence of drug-related severe liver dysfunction rarely occurs (<1 %) but is one of the serious adverse events by sorafenib. The authors highlight the case of a 71-year-old man with metastatic HCC with sorafenib-related fatal liver dysfunction (T-Bil 28.6 mg/dL, AST 1611 IU/L, ALT 1098 IU/L) 2 months later even without either intrahepatic viable HCC or hepatitis B virus (HBV) reactivation. Then, the liver dysfunction was improved following aggressive treatment using hyperbaric oxygen. A liver biopsy demonstrated cholestasis, degeneration, and necrosis in hepatocytes with lymphocyte infiltration. Thus, sorafenib rarely can induce liver dysfunction characterized by cholestatic and hepatocellular injury types, and it could be a fatal event. Clinicians should pay attention to any increase in the liver enzymes in these patients.

## Background

Sorafenib is an oral multikinase inhibitor and additionally inhibits VEGF and PDGF and affect as an anti-angiogenic agent, so it is worldwidely used for the treatment of advanced or metastatic hepatocellular carcinoma (HCC). While the two phase-3 randomized double-blind controlled trials (the Sorafenib Hepatocellular Carcinoma Assessment Randomized Protocol (SHARP) and Asia-Pacific trials) demonstrated statistically significant improvement in overall survival and in time to disease progression in patients with advanced HCC, the overall incidence of treatment-emergent (drug- or non-drug related) adverse events of any grades has been reported to be up to 81.9~97.2 % [1, 2]. The adverse events of higher frequency in sorafenib were hand-foot skin reaction, diarrhea, general fatigue, and anorexia; each frequency of which were reported to be 21–67, 11–39, 22–30, and 14–16 %. Of all the adverse events, the incidence of drug-related severe adverse events was 8.7 %, and liver dysfunction is rare but one of the major serious adverse events by sorafenib. Here, we showed the successfully treated case with fatal liver dysfunction induced by sorafenib.

## Case presentation

**Fig. 1:**
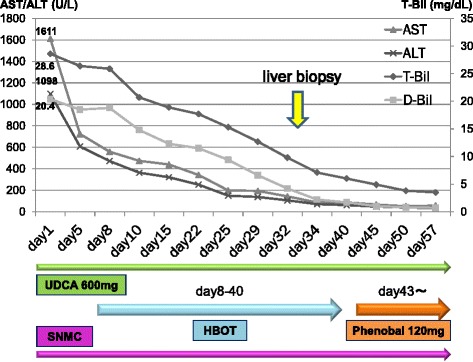
Time course of treatment and improvement of the liver dysfunction. *AST* aspartate aminotransferase, *ALT* alanine aminotransferase, *T-Bil* total bilirubin, *D-Bil* direct bilirubin, *UDCA* ursodeoxycholic acid, *HBOT* hyperbaric oxygen therapy, *SNMC* stronger neo-minophagen C

**Fig. 2:**
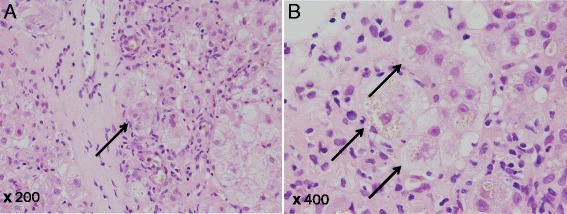
Pathological findings of sorafenib-induced liver dysfunction revealed by needle biopsy. **a** There are fibrous thickening in Glisson’s capsules, moderate P-P bridging fibrosis, piecemeal necrosis, and ridging necrosis in part. **b** There are moderate hepatocytic degeneration and necrosis, lymphocytic infilterate to Glisson’s cupsule, hepatocytic balooning, and cholestasis in hepatocyte

A 77-year-old man, with well-controlled hepatitis B virus (HBV) by entecavir (HBsAg positive, HBsAb negative, HBcAb positive, HBeAg negative, and undetectable HBV-DNA), was diagnosed with a primary HCC and was treated with radiofrequency ablation (RFA) in January 2010 at the previous hospital. Ten months later, he was admitted to our hospital because of the intrahepatic recurrences and then treated with RFA and with partial hepatectomy. One year later, he had a recurrence at the needle site of RFA on the chest wall and local resection was performed. However, he showed multiple and bilateral lung metastases with local recurrence at the chest wall, whereas there was no viable lesion in the liver. At that time, liver function was categorized as Child-Pugh class A with normal range of blood counts, electrolytes, and renal functions. Sorafenib was introduced 400 mg twice a day (total 800 mg/day), and there was no significant adverse event 1 week after the administration. Just after 1 month, when we checked, he is in a good general condition only with slight hand rubefaction that does not influence the treatment continuation and his laboratory data are without unexpected values (the serum total bilirubin 1.4 mg/dl, AST 33 IU/L, ALT 24 IU/L); he had general fatigue and appetite loss, and those symptoms gradually worsened despite improvement of the skin reaction. Two months after the induction of sorafenib, the symptoms aggressively worsened, and the laboratory data demonstrated significantly worsened liver functions in the serum bilirubin value (total bilirubin 28.6 mg/dl, direct bilirubin 20.4 mg/dl), transaminase values (AST 1611 IU/L, ALT 1098 IU/L), and other liver enzymes (Table 1), without significant evidence of HBV reactivation. By computed tomography, there is no significant finding such as intra- and extra-biliary duct dilatation and intrahepatic recurrence. Therefore, the fatal liver dysfunction was diagnosed due to sorafenib-related adverse event. We stopped the intake of sorafenib and treated with stronger Neo-Minophagen C and ursodeoxycholic acid. While serum transaminase values decreased in response to the above treatments, the serum bilirubin level did not change. As a next therapeutic approach, we started hyperbaric oxygen therapy (HBOT) every business day from day 8. HBOT was provided with a monoplace hyperbaric chamber. He was accommodated for about an hour and a half in the chamber, where the ambient pressure with pure oxygen increased two times higher than the local atmospheric pressure in 10 to 15 min; it was maintained for 60 min and was relieved back to the atmospheric pressure gradually in the same or more time spent than the increasing time. Thereafter, the serum bilirubin level started to decrease depending on the number of times of HBOT, and he could sustain himself with oral take (Fig. 1). Interestingly, the serum bilirubin levels failed to decrease during the weekend without HBOT. For example, while gradual decrease of the serum total bilirubin level was observed in the interval only on weekdays between days 15 (18.9 mg/dl) and 18 (17.4 mg/dl), there is no change of the bilirubin level in the interval including a weekend between days 18 and 22 (17.7 mg/dl). This tendency was observed during the prominent hyperbilirubinemia. A liver biopsy was performed, and the microscopic findings demonstrated cholestasis, degeneration, and necrosis in hepatocytes with lymphocyte infiltration (Fig. 2). On day 43 from the start of treatment, although a phenobarbital was administered in addition to HBOT in anticipation of further improvement in bilirubin level, an additional phenobarbital treatment seemed to be not effective. After 2 months of treatment, serum bilirubin level was around 3 mg/dl and he was discharged and restarted to treat HCC with tegafur/uracil leucovorin, but as progression of the lung metastases, he died 1 year after discharge.

**Table 1:** Laboratory data on admission

Total-bilirubin	28.6 mg/dl	α-fetoprotein (AFP)	98.2 ng/ml	PT	16.3 s
D-Bilirubin	20.4 mg/dl	AFP-L3	20.1 %	PT	60 %
Albumin	3.3 g/dl	PIVKA-II	876 mAU/ml	PT	1.47
ALT	1098 U/l	CEA	3 ng/ml	APTT	39.4 s
AST	1611 U/l			APTT	67 %
Ammonia	29 μg/dl	White blood cell	4300/μl		
Bile acid	175 μmol/l	Neutrocytes	68 %		
γ-GTP	136 mg/dl	Hemoglobin	12.7 g/dl		
LDH	70 U/l	Platelet counts	7.3 × 10^3^/μl		
ALP	606 U/l	Child-Pugh score	8 (grade B)		
Cholinesterase	116 U/l	MELD score	23		
CRP	3.09 mg/dl				
BUN	12.8 mg/dl				
Creatinine	0.85 mg/dl				
PT	60 %				
PT-INR	1.47				

*ALT* alanine transaminase, *AST* aspartate aminotransferase, *γ-GTP* γ-glutamyl transpeptidase, *LDH* lactate dehydrogenase, *ALP* alkaline phosphatase, *CRP* C-reactive protein, *BUN* blood urea nitrogen, *PT* prothrombin time, *PT-INR* prothrombin time-international normalized ratio, *PIVKA-II* des-γ-carboxy prothrombin, *CEA* carcinoembryonic antigen

## Conclusions

Here, we showed the metastatic HCC case with fatal liver dysfunction induced by sorafenib which successfully improved following aggressive treatment using HBOT. The presented case revealed a conjugated hyperbilirubinemia and liver injury even without intrahepatic viable HCC. Indeed, liver biopsy proved the cholestasis and liver damage in hepatocytes with lymphocyte infiltration due to sorafenib intakes. In addition, the fact that HBOT was successful in the presented case suggests the type of liver injury induced by sorafenib. The benefits of HBOT against cholestasis have been reported in vivo [3, 4], and the mechanism of the therapeutic effect of HBOT to liver injury is reported to consist of hepatoprotection associated with anti-oxidative potential and promotion of liver regeneration [5]. Thus, sorafenib-induced liver dysfunction may be characterized by cholestasis and cell deaths in hepatocytes with inflammatory cell infiltration.

The most of sorafenib-associated adverse effects are mild to moderate and tolerable [2, 6]. On the other hand, unexpected and serious toxicities including liver injury have also been reported. In the many clinical settings, it is difficult to judge whether liver injury is caused by drug toxicity or disease progression in patients with HCC. Fortunately in this case, there was no viable HCC in the liver, and no evidence of reactivation of HBV. These backgrounds greatly helped to diagnose the liver dysfunction as a sorafenib-induced liver injury. In the phase I study of sorafenib for patients with advanced, refractory solid tumors, steady-state plasma concentrations were reached within 7 days by twice-daily oral intake [7]. In this study, transient grade 3 elevation of conjugated bilirubin, without concomitant elevation of other hepatic enzymes, was reported in 3 (4 %) of 69 patients, and it was described that the elevation of bilirubin appeared independently of the dose level and occurred on day 3 after the first application and resolved spontaneously by day 5 [7]. In renal cell carcinoma patients, sorafenib-related serious adverse event was found at 10.7 %. Of them, the most frequently occurring drug-related serious treatment-emergent adverse events were liver dysfunction, aspartate aminotransferase elevation, and alanine aminotransferase elevation, each of which was reported at 2.3 % [8]. In advanced HCC, the difference of sorafenib pharmacokinetics between Child-Pugh A and B patients were not considered clinically significant. As a mechanism of sorafenib-induced liver injury, metabolic dysfunction has been reported [9]. Sorafenib requires glucuronidation catalyzed by uridine diphosphate glucuronosyltransferase (UGT) 1A9 of oxidized sorafenib metabolites formed through cytochrome P450 (CYP) 3A4 metabolism. For example, sorafenib exposure was reduced by an average 37 % with concomitant administration of the CYP3A4 inducer rifampicin (rifampin); sorafenib concentrations may also be decreased by the other CYP3A4 inducers [9]. These enzymes show phenotypic variability based on polymorphism, suggesting sorafnib metabolism may be related with polymorphisms of the above enzymes, though the polymorphism in the present case were unexamined. Similarly, sorafenib inhibits UGT1A1 bilirubin conjugation and phenotypically induce hyperbilirubinemia in patients with Gilbert’s syndrome [10]. In this case, prolonged hyperbilirubinemia was so outstanding, and histologically, cholestasis is not in the biliary tract but mainly in hepatocyte that sorafenib might have inhibit the intracellular process of bilirubin conjugation. In future investigations, the exploration of polymorphism in some enzymes such as CYP3A4 and UGT1A9 may be useful to predict the sorafnib-induced liver dysfunction.

Sorafenib rarely can induce liver dysfunction characterized by cholestatic and hepatocellular injury types, and it could be a fatal event. We advocate appropriate liver function surveillance in patients with sorafenib, and the presented HBOT was effective for sorafenib-induced cholestasis.

## Consent

Written informed consent was obtained from the patient for publication of this case report and any accompanying images.

## Abbreviations

CYP: cytochrome P450
HBV: hepatitis B virus
HCC: hepatocellular carcinoma
RFA: radiofrequency ablation
UGT1A9: uridine diphosphate glucuronosyltransferase 1A9
